# Experimental Study on the Mechanical Performance of Reinforced Concrete Joints Connected by T-Shaped Steel Plates

**DOI:** 10.3390/ma18030600

**Published:** 2025-01-28

**Authors:** Jian Wu, Ying Jiang, Jian Zhou, Chaoqun Hu, Jianhui Wang, Weigao Ding

**Affiliations:** 1 Shaanxi Key Laboratory of Safety and Durability of Concrete Structures, Xijing University, Xi’an 710123, China; wujian2085@126.com (J.W.);; 2 Northwest Engineering Co., Ltd., Xi’an 710065, China; 3 School of Infrastructure Engineering, Dalian University of Technology, Dalian 116024, China

**Keywords:** reinforced concrete joints, T-shaped steel plate, experimental study, mechanical performance

## Abstract

In the case of engineering structures, the performance of a structure will gradually deteriorate with an increase in the usage time, leading to a decrease in the safety of the structure. In addition, even if the safety of a structure is reliable, its current structure type may no longer meet the latest usage requirements. Therefore, four reinforced concrete specimens were produced in this study: one was a cast-in-place specimen, and three were specimens connected by a T-shaped steel plate with steel cladding reinforcement. This article first introduces the structural form and construction method of the new types of joints, and then it describes the quasi-static testing that was conducted to analyze seismic performance indicators such as the failure characteristics, bearing capacity, ductility, stiffness degradation, and energy dissipation. Finally, combined with a strain analysis of the steel bars and steel plates, the force transmission mechanism of the new types of joints was investigated. The research content of this paper helps to promote the progress of structural retrofitting and strengthening work and the sustainable development of the construction industry.

## 1. Introduction

The reinforced concrete structure is a widely used structural form, and most of the existing buildings in Chinese cities are reinforced concrete frame structures [1]. However, due to the aging of the material itself, external environmental effects, and seismic effects, this type of structure will inevitably experience performance degradation, endangering the normal use and safety of the structure. At the same time, part of the structure, even if it is still within the normal service life time, may need to be modified to meet new use requirements [2,3]. Therefore, it is of high practical engineering significance to strengthen and renovate existing reinforced concrete structures.

How to add new components to existing components and through reliable connections to meet the requirements for the load-carrying capacity and seismic performance of the components or structures is a hot issue in current research. At present, the main connection methods used are reinforcement connections, bolt connections, and welding connections. Nouri et al. [4,5] used mechanically anchored reinforcement bars for the connections between steel column flanges and cross-laminated timber (CLT) floor slabs. The results showed that the implanted connections were able to effectively transfer the tension forces generated in the CLT floor slabs, thus significantly enhancing the stiffness and moment load capacity of the beam–column connections. Wiktor et al. [6] investigated the flexural performance of combined nodes in timber structures with implanted connections, and it was found that this type of connection not only improved the compressive and shear performance of the structure but also maintained a stable performance under a wide range of environmental conditions. Ozturan et al. [7] conducted repeated cyclic loading tests on specimens with three different connection types: cast-in-place, welded, and bolted connections. The results showed that bolted connections, when optimized, had a higher energy dissipation capacity and better fabrication convenience, which made them more applicable in earthquake-prone regions. Lee et al. [8] made five beam–column joints connected using bolting and prestressing methods. The test results showed that the specimens made using this connection method could effectively transfer loads under seismic action, but their energy dissipation capacity was slightly insufficient. Baran et al. [9] proposed an innovative beam–column node connection with pre-installed screws in the columns and pre-installed steel connection plates at the beam ends, and then the beams were connected to the columns by bolts and angles. It was shown that the damage mainly occurred at the beam end and in the connection region, while the damage to the column was relatively small. Ding et al. [10] designed an assembled beam–column joint with high-strength bolt connections. They found that the joint connected with high-strength bolts had a better yield capacity, ultimate capacity, initial stiffness, and energy dissipation performance than the specimens connected with low-strength bolts. Tnakut et al. [11] investigated the seismic performance of cast-in-place beam–column joints and welded steel plate connection joints, and these two types of joints were similar in terms of their strength, stiffness, ductility, and other performance indicators. Rodríguez et al. [12] proposed a connection method using welded reinforcement to connect precast concrete members and found that the welded joints had poor energy dissipation capacities and ductile properties, and the reinforcement welds were prone to brittle damage. Magliulo [13] took the improvement in the seismic performance as a starting point for the design of new connection methods for beam–column joints, and from the experimental results, it could be seen that the quality and location of the weld had a greater impact on the performance of specimens. Esmaeili et al. [14] designed a joint with welded connections through steel plates to steel cylinders inside precast columns, which had the advantages of high strength and reliability to ensure the safety of precast concrete structures under seismic loads. In addition, other studies [15,16,17] also investigated the mechanical performance of precast or assembled RC column–beam joints using bolts and steel plates. The results showed that this connection method could ensure the safety of the structure. However, the construction processes for this method are complex, and the cost is high, which is not conducive to its popularization and application. Therefore, compared with bolt connections and planting connections, components with welded connections show better performance and can ensure structural integrity, so this paper describes the welding of a T-shaped steel plate onto existing columns, followed by the welding of the reinforcement of the beams to the flanges of the T-shaped steel plate to enhance the integrity of the structure.

In a reinforcement and renovation project, in order to meet the requirements for existing buildings for the transformation of beam–column joints or the repair of damaged beam–column joints, it is necessary to carry out reinforcement treatments through certain structural measures, and the commonly used methods of reinforcement are the enlarging section method, wrapped steel method, carbon fiber-reinforced plastic (CFRP) fiber reinforcement method, and steel angle reinforcement method. Compared with the CFRP fiber reinforcement method and enlarging section method, the steel reinforcement method does not affect the service space of the structure, and the materials used can withstand tensile and compressive loads. Vandoros et al. [18] investigated the effect of the enlarging section method on the mechanical performance of the column. It could be observed that overall performance of the column strengthened using different enlarging section methods was significantly improved. Guo [19] used the ANSYS software to simulate the mechanical properties of the structure after strengthening using the enlarging section method, and the results showed that this method could significantly improve the bearing capacity and seismic performance of frame structure building. Martin et al. [20] studied the mechanical properties of RC columns strengthened using the fiber concrete enlarging section method. The results showed that the use of the enlarging section method could be able to effectively reduce crack width and significantly enhance bearing capacity. Kim et al. [21,22] treated reinforced concrete beams and columns using the wrapped steel method, which gave full play to the properties of the steel and improved the mechanical properties of components. Xu et al. [23] designed four seismically damaged steel reinforced concrete columns and carried out numerical simulations with ABAQUS software. The test results showed that the columns could meet requirements of strong columns and weak beams in seismic design. Chang-Geun et al. [24] conducted an experimental study on the seismic performance of reinforced concrete beam–column joints using embedded carbon fiber reinforcement and CFRP. The experimental study showed that this method significantly reduced the damage to joints, thus improving the mechanical performance of the structure. Beydokhti et al. [25] carried out post-damage strengthening tests of CFRP strengthened beam–column joints. It could be concluded that CFRP could significantly improve the seismic performance of the joints, but when the damage exceeded a certain limit, the repair effect of CFRP would no longer be obvious. Truong et al. [26] strengthened the beam–column joints using reinforcement anchorage, CFRP wrapping, and wrapped steel. It could be observed that all the schemes improved the seismic performance of the joints. Azarm et al. [27] strengthened the beam–column joints using CFRP. The results showed that CFRP could effectively improve the bearing capacity of the joints. Also, the steel angle was another reinforcement method to improve the performance of structures [28,29,30,31]. Although these reinforcement methods can greatly improve the mechanical performance of the structure or components, the enlarging section method is easy to affect the use of space of buildings, while CFRP or the steel angle reinforcement method in a complex structure of the site is not easy to construct. Therefore, this paper chooses to use the wrapped steel method for the reinforcement of joint.

A new type of the column-beam joint that connects new beams to existing columns is designed to investigate the renovation and reinforcement of buildings in this paper. When making the specimen, cast-in situ column is firstly made, then the column is wrapped with steel plates and T-shaped steel plates are welded on both sides of the wrapped steel, then the longitudinal reinforcements of the beam are welded onto the flanges of T-shaped steel plates and the concrete of the beam is poured. The wrapped steel of beams and columns are connected together by welding, and the steel plates are connected to the concrete by chemical anchor bolts and bolts. The mechanical characteristics of the new-type specimen is analyzed through the low cyclic loading test, and seismic performance indexes such as hysteresis curves, skeleton curves, ductility, and energy dissipation capacity are investigated to promote the promotion and application of this construction form.

## 2. Design and Specimen Making

### 2.1. Designing of Specimens

**Figure 1:**
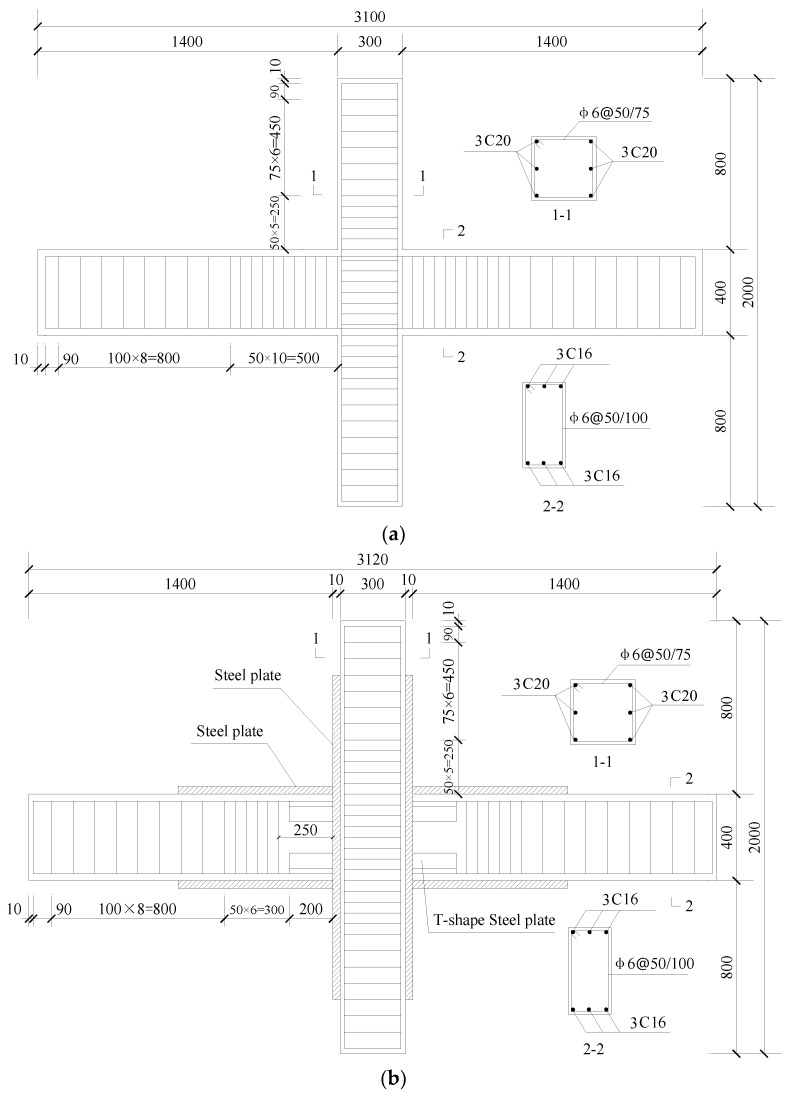
The dimension and reinforcement condition of specimens: (**a**) RC0 and (**b**) TRC1–TRC3.

Four specimens were designed in this paper, one cast-in situ specimen (RC-0) and three specimens connected by a T-shaped steel plate (TCR1–TCR3, new-type specimen), both of which had the same dimensions. The parameter and arrangement of materials used in this paper are given in Table 1. The dimensions of specimens can be seen in Figure 1.

**Figure 2:**
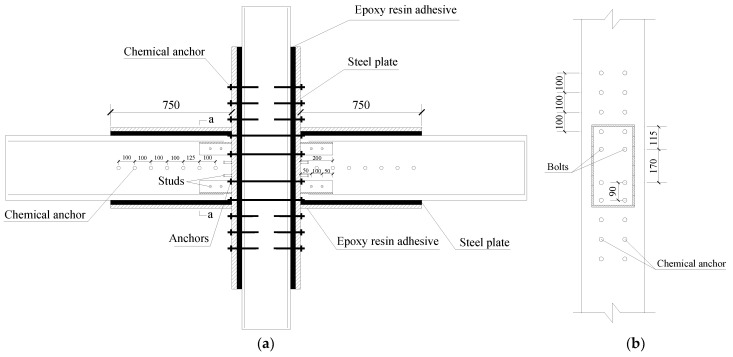
Design drawings of specimens: (**a**) sectional drawing and (**b**) side view.

**Figure 3:**
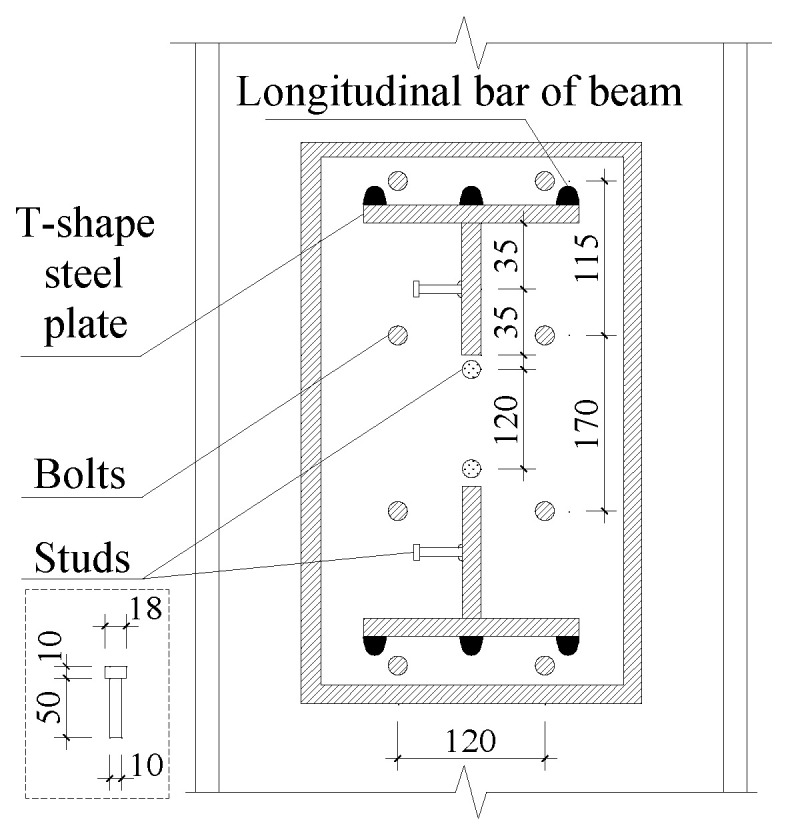
An a–a section view.

TRC1–TRC3 take the thickness of the steel plate wrapping the beam and the T-shaped steel plate as the parameter variables. The dimensions of the T-shaped steel plate (flange width×web height×plate thickness) of TRC1, TRC2, and TRC3 are 200 mm × 70 mm × 5 mm, 200 mm × 150 mm × 5 mm, and 200 mm × 150 mm × 3 mm, respectively. The thickness of the wrapped steel of the beam of TRC1, TRC2, and TRC3 are 3 mm, 2 mm, and 3 mm, respectively. While for column, the thickness of the wrapped steel plate is 10 mm. The strength grade of the steel plate is Q345. The designing condition of the new-type joint can be seen in Table 2 and Figure 2 and Figure 3.

As shown in Figure 2a, a layer of epoxy resin adhesive is applied to the surface of the concrete before the wrapping steel treatment of the specimen. To strengthen the interaction between the steel plate and concrete, chemical anchors are arranged on the surface of the specimen, which could be seen in Figure 2a,b. The strength grade and diameter of the chemical anchors are 5.8 and 8 mm, respectively. It should be noted that considering the existence of lateral reinforcement, the distance between the chemical anchors on the beam has a value of 125 mm, while for the chemical anchors on the column, the spacing is 100 mm. The stress of the core area of joint is complex. Therefore, in order to better realize the effective connection of the beam and column, bolts, which have a strength grade of 8.8, a diameter of 12 mm, and a length of 350 mm, are used to reinforce the connection between the steel plate and column. Considering the weak bonding effect between the concrete and the steel plate, studs are welded onto the wrapped steel of the column and the web of the T-shaped steel plate, as shown in Figure 3. The strength grade of studs is 10.9, and the dimension of the studs could also be seen in Figure 3. The connection of the steel plates is made by groove welding, and the studs are connected to the steel plates by stud welding. All the weld grades are grade one.

### 2.2. Manufacture of Specimens

RC0 is a one-time casting pouring, while TRC1–TRC3 are made in two steps, the first step is the casting of the columns, after the columns have been cured for 28 d and the wrapped steel has been conducted, the beam concrete is cast and the wrapping steel treatment is carried out (second step).

The steps of the specimen RC0 production are pasting strain gauges → tying the reinforcement cage → erection of shuttering → pouring concrete → specimen curing.

**Figure 4:**
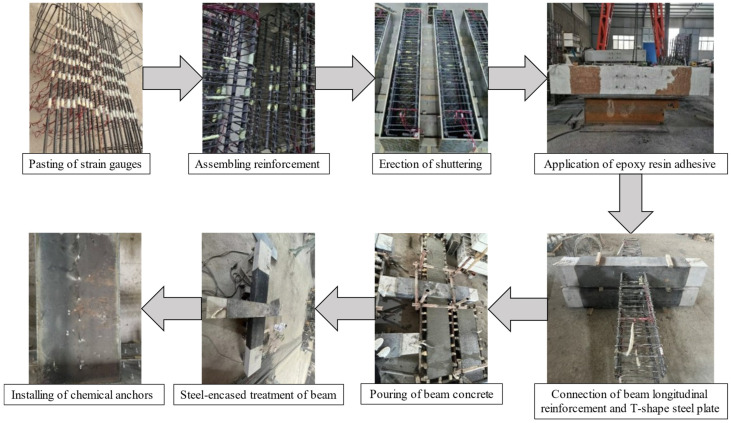
Manufacture of TRC1–TRC3.

The fabrication of specimens TRC1–TRC3 is divided into the following steps: Pasting strain gauges → tying the reinforcement cage of the column → erection of the shuttering of the column → pouring the concrete of the column → wrapping the column with steel and welding the T-shaped steel plate → erection of the shuttering of the beam → pouring the concrete of the beam → wrapping the beam with steel → installing the chemical anchor and bolts, as shown in Figure 4.

### 2.3. Materials Properties

#### 2.3.1. Concrete

**Figure 5:**
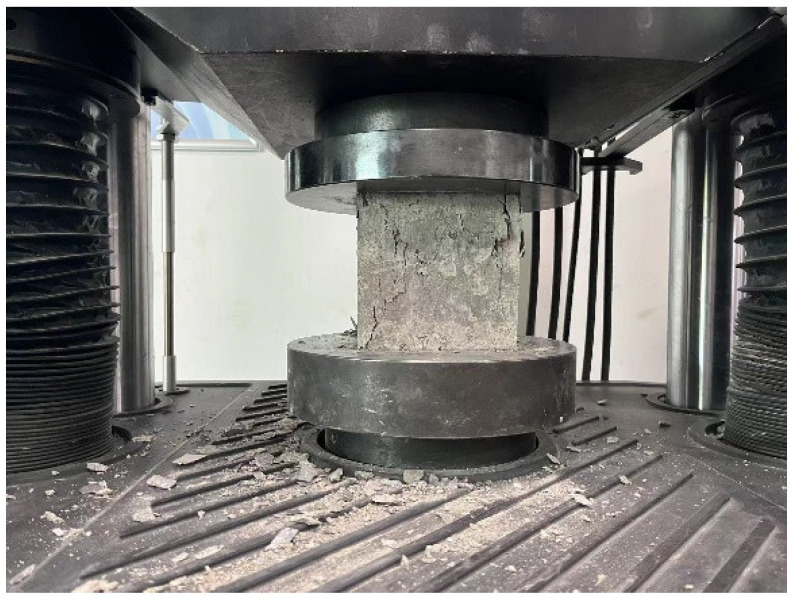
Failure mode of cube specimens.

This test contains two different strength grades of concrete (C40 and C50), where C40 is used for the beam and C50 is used for the column. Based on the aforementioned specimen production process, the reserved concrete cube are divided into three groups, each of which contains three specimens. The first group is the concrete reserved when pouring the beam of RC0 (C40), the second group is the concrete reserved when pouring the column (C50), and the third group is the concrete reserved when pouring the beam of TRC1–TRC3 (C40-1). According to the provisions of Chinese standard GB/T 50081-2019 [32], the size of the cubic specimen is 150 mm × 150 mm × 150 mm, and the loading speed is 0.6 MPa/s. The failure phenomenon of the specimen is shown in Figure 5, and the test results are given in Table 3.

#### 2.3.2. Steel Bars and Steel Plates

**Figure 6:**
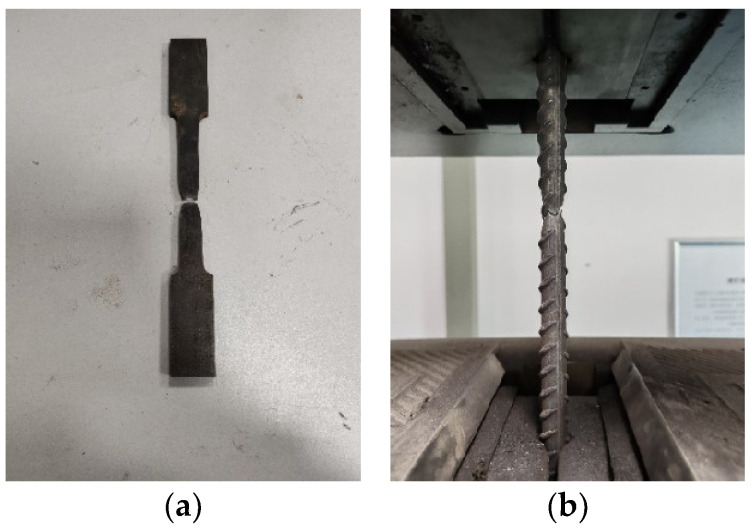
Failure mode of (**a**) the steel plate and (**b**) the steel bar.

The tensile strength tests of the steel bars and plates are carried out according to Chinese standard GB/T 228.1-2010 [33]. Three specimens of each type of the steel bar and plate are taken as a group, and the tensile strength test is carried out to determine the yield strength and ultimate strength of the steel using the MTS 2000 kN universal testing machine with a loading speed of 1.2 kN/s. Figure 6 shows the failure phenomenon of the steel, and Table 4 gives the test results.

### 2.4. Testing Methods

**Figure 7:**
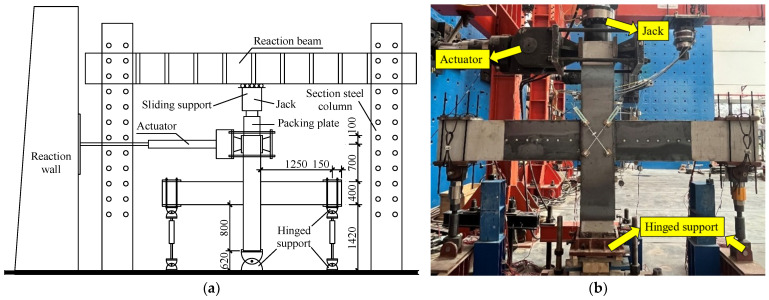
Loading device of the testing method: (**a**) schematic diagram; (**b**) actual loading diagram.

The loading device is shown in Figure 7. Considering the mechanical characteristics of RC structures in actual projects, hinged support is used at the end of the beam and the lower end of the column. The horizontal load is provided by an MTS actuator with a maximum load of 100 t, and the vertical load is applied by a 200 t hydraulic jack.

**Figure 8:**
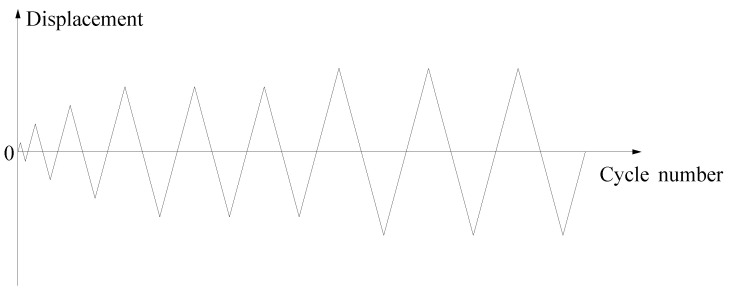
Loading scheme of the testing method.

In the loading process, the vertical load is firstly applied slowly to a set value of 400 kN, and the stability of this value is monitored during the test to ensure that the axial pressure ratio is 0.19. Then, the horizontal load is applied, and it should be specified that the actuator is pushed in the positive direction and pulled in the negative direction. According to the provisions of Chinese standard JGJ/T 101-2015 [34], the displacement control method is used for loading. Cyclic loading is loaded step by step with an increase of 3 mm before the yielding of the specimen, and each level is cycled once. After the yielding of the specimen, the cast-in situ specimen and the new-type specimen is loaded step by step with an increase of 21 mm and 15 mm, respectively, and each level is cycled three times. The loading program has been given in Figure 8. When one of the following conditions is met, it is judged that the specimen is damaged: (1) large deformation occurs in the wrapped steel of the beam or column; (2) through cracks appear in the weld joints of the wrapped steel; (3) large lateral displacement occurs in the specimen; (4) the load carrying capacity decreases to approximately 85% of maximum load carrying capacity.

## 3. Analysis of the Experimental Process and Failure Characteristics

Considering that the loading scheme used in this paper is the displacement control method, displacement is used as the dividing criterion in the description of failure process of the specimen.

### 3.1. Process of the Specimen Failure

(1) RC0

- 12 mm: Diagonal cracks appeared on the left side beam.
- 15 mm: The sound of concrete being squeezed and cracked could be heard, four vertical cracks could be observed on the left beam, and two vertical cracks appeared on the right beam with a length of 20 cm.
- 18 mm: Transverse cracks appeared in the lower column, approximately 20 cm long. Two cracks with a length of 10 cm could be seen on the right beam, and the existing cracks on the left beam continued to develop.
- 21 mm: A new diagonal crack approximately 10 cm long appeared in the right beam.
- 63 mm: A large number of bending cracks appeared at the beam end near the core area, and a number of vertical cracks could be observed on the right side of core area, forming a web-like crack distribution.
- 84 mm: No new cracks appeared on the beam, and the main diagonal crack could be seen in the core area. The concrete at the beam end close to the core area was cracked, and a small amount of concrete fell off.
- 105 mm: Diagonal cracks in the core area were increasing in width, and the concrete of the beam close to the core area was crushed.
- 126 mm: The falling off of concrete led to the exposure of hoop longitudinal bars, and the specimen was severely damaged.

**Figure 9:**
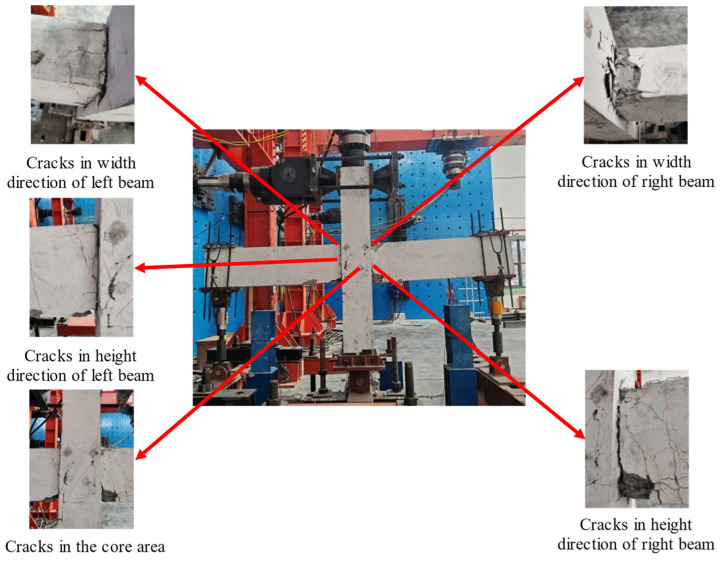
The failure phenomenon of RC0.

The overall and local damage patterns of the specimen are shown in Figure 9.

(2) TRC1

- 9 mm: The sound produced by the deformation of epoxy resin adhesive between the wrapped steel and concrete could be heard.
- 12 mm: The sound of cracking concrete could be heard, and vertical cracks appeared at the left beam with a length of 5 cm.
- 15 mm: Two cracks with a length of 15 cm appeared in the concrete of left beam.
- 24 mm: The wrapped steel and the reinforcement of the beam reached yield strength.
- 39 mm: New diagonal cracks appeared in the beam concrete and bulging could be observed on the wrapped steel of beam.
- 54 mm: Cracks appeared in the weld of the wrapped steel around the beams and columns.
- 69 mm: The weld cracks on steel plates wrapped around the beam and column were gradually developing.
- 84 mm: The bulging of the wrapped steel on the beam became more significant, and the length of weld cracks between the wrapped steel of the beam and column was 8 cm in the direction of the beam height and 5 cm in the direction of the beam width.
- 99 mm: Weld cracks appeared on the wrapped steel of the column.
- 114 mm: The anchor bolts were visible from the crack in the weld seam at the lower end of left beam, and the crack in the weld seam of the wrapped steel of the beam and column ran through in the width direction of beam.
- 129 mm: The weld cracking of steel plates on both sides of the column continued to develop, and the deformation of steel plates was significant. At this time, the load was 91.3 kN, which had decreased to below 85% of its ultimate load, indicating that the specimen was damaged.

**Figure 10:**
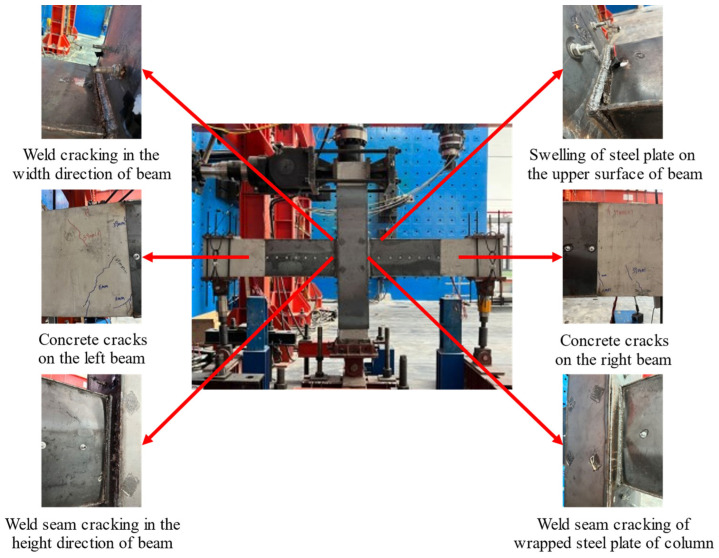
The failure phenomenon of TRC1.

The overall and local damage patterns of the specimen are shown in Figure 10.

(3) TRC2

- 9 mm: The sound of cracking concrete could be heard, and a vertical crack with a length of 5 cm appeared underneath the concrete of left beam.
- 15 mm: There was a tearing sound of epoxy resin adhesive between the wrapped steel and concrete, and the adhesive cracking could be seen from the edge of the wrapped steel.
- 21 mm: The chemical anchor bolts arranged on the steel plates on both sides of the column loosened and the nuts were extruded outwards. At the same time, three vertical cracks were added to the beam concrete.
- 24 mm: The outer steel plate and longitudinal reinforcement of the beam yielded and the specimen was in the yield state.
- 39 mm: Bulging began to appear on the wrapped steel of beam, and the crushing sound of concrete could be heard during the loading process.
- 54 mm: A crack approximately 2 mm long appeared at the corner of weld between the beam and column.
- 69 mm: The bulging in the beam height direction became larger, and the cracks in the connection welds of the beam and column gradually developed along the beam width and beam height direction.
- 84 mm: The length of cracks at the welding of beams and columns was 5 cm along the beam height direction, and the length of cracks along the beam width direction was 6 cm.
- 99 mm: A small crack of approximately 6 cm long appeared at the weld seam of the wrapped steel around the column.
- 114 mm: The wrapped steel of the column was torn at the weld seam, and the cracks at the welding point of the beam and column penetrated in the width direction of the beam.
- 129 mm: The weld seam of the wrapped steel of the column was almost cracked and penetrated in the core area. At this time, the steel plate broke, the deformation of the steel plate was more serious, and the load dropped to 89 kN, and the specimen was judged to be damaged.

**Figure 11:**
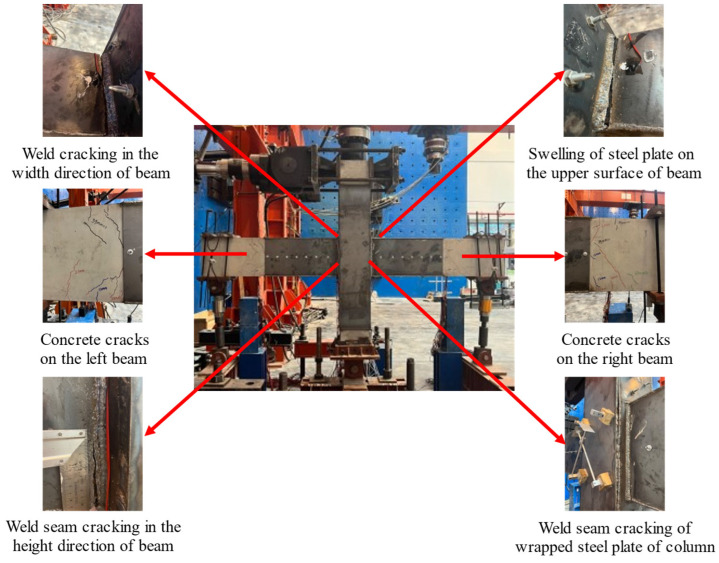
The failure phenomenon of TRC2.

The overall and local damage patterns of the specimen are shown in Figure 11.

(4) TRC3

- 9 mm: A small crack appeared in the concrete of left beam.
- 12 mm: The concrete fell off from the surface of left beam, and the sound of crushing concrete could be heard. A 10 cm long vertical crack appeared on the concrete of right beam.
- 15 mm: The cracking of epoxy adhesive could be seen from the edge of the wrapped steel and a diagonal crack of 6 cm in length appeared on the concrete.
- 18 mm: The cracking sound of concrete could be heard, and new cracks appeared in the concrete of beams.
- 21 mm: The chemical anchors arranged on the wrapped steel of the column became loose and the nuts were squeezed outwards.
- 24 mm: Fine cracks started to appear at the corners of the weld seam connecting the wrapped steel of the beam and column. The steel plates surrounding the beam and longitudinal bars began to yield, indicating that the specimen has reached the yielding state.
- 39 mm: Multiple cracks appeared on the concrete of beam, accompanied by the sound of concrete cracking. The nuts of chemical anchors on the column were compressed outward by approximately 3 mm.
- 54 mm: A vertical crack appeared in the concrete of left beam, and a slight bulging in the upper part of the wrapped steel of beam. The weld seam at the corner of the wrapped steel around the beam and column cracked.
- 69 mm: The crack length of the weld seam at the corner of the wrapped steel around the beam and column was approximately 2 cm, and the bulging on the upper part of the wrapped steel increased.
- 84 mm: The bulging in the direction of the beam width and height was aggravated, and the cracks at the corners of the beam continued to develop.
- 99 mm: Fine cracks began to appear at the weld seam of the wrapped steel on both sides of the column.
- 114 mm: The wrapped steel of the column cracked at the weld seam, and the weld seam between the left beam and the lower side of the column cracked and penetrated.
- 129 mm: It could be seen that the bolts fixing the wrapped steel of the column had been broken, and the cracks had penetrated the upper and lower welds of the beam and column. The welds of the wrapped steel outside the right column were cracked and penetrated in the core area. At this point, the load dropped to 82 kN, indicating that the specimen had been failed.

**Figure 12:**
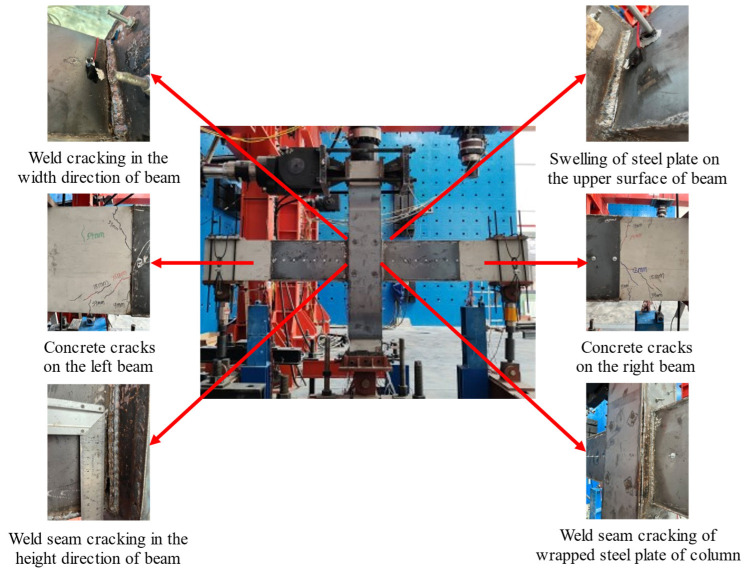
The failure phenomenon of TRC3.

The overall and local failure modes of the specimens are shown in Figure 12.

### 3.2. Failure Characteristics Analysis

**Figure 13:**
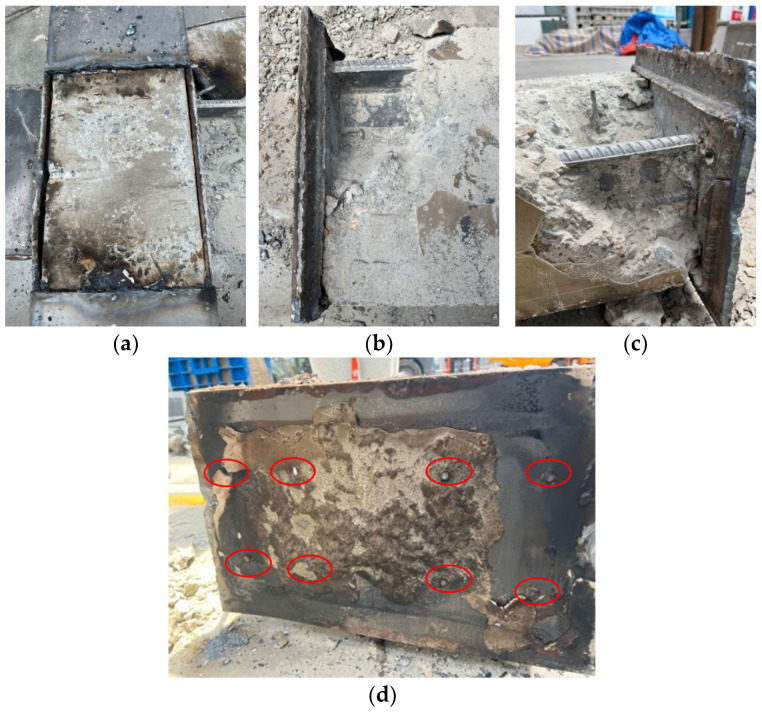
The failure phenomenon of the core area of the specimen: (**a**) concrete in the core area; (**b**) web of the T-shaped steel plate; (**c**) flange of the T-shaped steel plate; (**d**) bolts used for the fixing of the steel jacket of the column.

RC0 and TRC1–TRC3 exhibit different characteristics during the destruction process:

(1) Comparing RC0 with TRC1–TRC3, it could be concluded that the cast-in situ specimen experienced typical shear damage in the core area of joint, while the damage to new-type specimens occurred mainly in the weld seam of the wrapped steel connecting the beam and column. After the experiment, when the steel plate in the core area was cut open, there were almost no cracks could be observed in the concrete of core area (Figure 13a). The main reason for this phenomenon is that the wrapped steel restricts the deformation of concrete in the core area, which improves its mechanical performance.
(2) Comparing the failure modes of TRC1 and TRC2, it could be found that the weld seam of the wrapped steel connecting the beam and column in TRC1 cracked almost completely along the beam height direction, while that of TRC2 cracked by 2/3 of the beam height, indicating that the thickness of the steel plate wrapping the beam had a certain influence on the failure mode of the specimen.
(3) Comparing the failure modes of TRC2 and TRC3, it could be found that the cracks width of the weld seam on the column side of TRC2 was slightly larger than that of TRC3. At the end of testing, the steel and concrete of the beam were cut open, as shown in Figure 13b–d. It could be found that the web plate (Figure 13b) and flange (Figure 13c) welded to the wrapped steel of the column had not been damaged, while all the bolts had broken (Figure 13d). The weld seam of the steel plate wrapping the column were damaged, indicating that the thickness of the connectors had little effect on the failure mode of the specimens. It should be noted that the red circles in Figure 13d were the fracture surface of the bolts.

## 4. Result Analysis of the Testing Method

### 4.1. Hysteresis Curves

**Figure 14:**
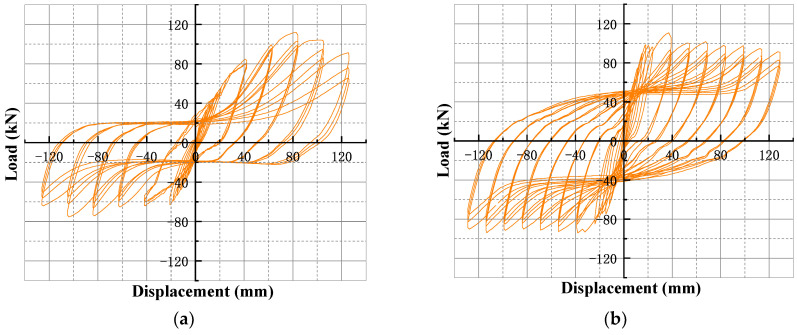

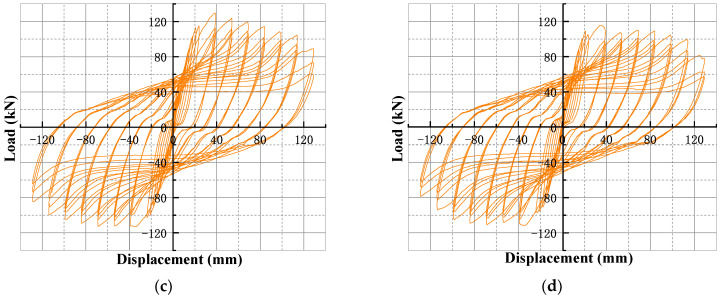
The hysteresis curve of (**a**) RC0, (**b**) TRC1, (**c**) TRC2, and (**d**) TRC3.

The hysteresis curve can reflect the deformation, stiffness degradation, and energy consumption of the structure during the process of repeated force, and the fuller the shape of the hysteresis curve, the stronger the ability of component to consume seismic energy. The hysteresis curves of joints are shown in Figure 14. It could be concluded from the curve shape that:

(1) At the beginning of loading, the specimen was in the elastic stage, the hysteresis loop was basically linear, the area of the hysteresis loop was very small, and the energy consumption was small.
(2) After the yielding of the specimen, the hysteresis curves gradually became full with the increasing of loading displacement, and the shape gradually changed to an inverse S-shape. The stiffness of the specimen gradually decreased, and the area of hysteresis loop gradually increased, reflecting an increase in the dissipation capacity of the specimen.
(3) After the specimen started the three cyclic loading process, the slope of hysteresis loop of the first cyclic loading was significantly larger than the corresponding values of the second and third, indicating that the damage to the specimen was gradually accumulated, which led to a reduction in its mechanical properties.
(4) After the loading displacement was greater than or equal to 99 mm, the peak point of hysteresis curves gradually decreased, and the slope of loading section also decreased. The shape of hysteresis loop gradually became Z shaped, and the pinching phenomenon was obvious.
(5) By comparing the hysteresis curves of RC0 and TRC1–TRC3, it could be concluded that the new-type specimen had a greater load-bearing capacity, which indicated that the wrapped steel and T-shaped steel could ensure the integrity of the specimen. While comparing the hysteresis curves of TRC1 and TRC2, it could be seen that the hysteresis loop of TRC2 was fuller and had a larger load-bearing capacity, indicating that increasing the thickness of the steel plate wrapping the beam could effectively increase the load-bearing capacity and energy dissipation capacity of the specimen. Comparing the hysteresis curves of TRC2 and TRC3, it could be found that the hysteresis curves of two specimens were basically the same, and the load-bearing capacity of TRC2 was slightly increased compared with that of TRC3, which indicated that the increase in the thickness of connecting parts of the beam–column joint was beneficial to improve the load-bearing capacity of the specimen, but the effect was not obvious.

### 4.2. Skeleton Curves

**Figure 15:**
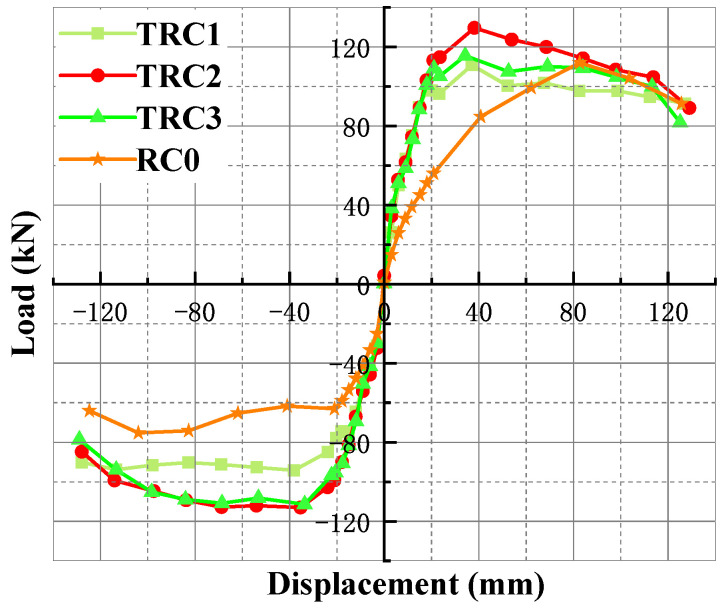
The skeleton curve of the specimens.

A skeleton curve is the outer envelope curve connected by the peak points of hysteresis curves, thus skeleton curves and hysteresis curves have a certain correspondence in shape, but the skeleton curve succinctly reflects the load-bearing capacity and stiffness characteristics of the structure or component. The skeleton curves of the specimen studied in this paper are shown in Figure 15.

(1) When the specimen had not yielded, the skeleton curves of the specimen were all straight, indicating that the specimen was in an elastic working state. After the yielding of the specimen, the slope of curve decreased with the increase in displacement. After reaching the maximum load, the skeleton curve began to decline, indicating the gradually loss of bearing capacity.
(2) Comparing the skeleton curves of TRC1 and TRC2, it could be seen that the thickness of the steel plate wrapping the beam had a significant effect on the improvement of load-bearing capacity of the joint. The stiffness of these two specimens was basically the same in the early stage of loading, while the bearing capacity of TRC2 decreased faster in the later loading period, and the high bearing capacity could not be maintained after the specimen was damaged. This was mainly due to the weld failure of the wrapped steel connecting the beam and the column, which weakened the integrality of the specimen and reduced the bearing capacity.
(3) Comparing the skeleton curves of TRC2 and TRC3, it could be seen that the shape difference of the skeleton curves of these two specimens was relatively small, but the load-bearing capacity of TRC2 was obviously larger than that of TRC3, which indicated that the increase in the thickness of the connector did not have much effect on the load-bearing capacity of the specimen.

### 4.3. Ductility Analysis

**Figure 16:**
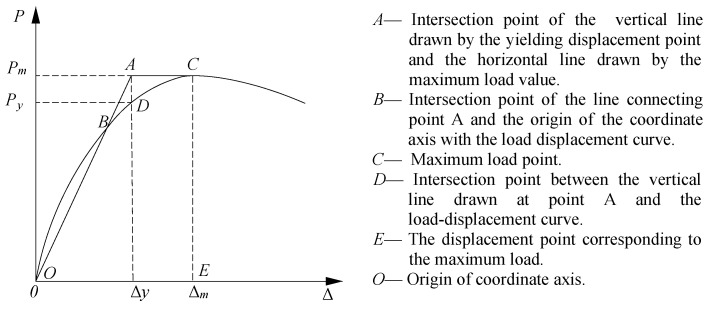
Schematic diagram of the equivalent energy method.

The yield point of the specimen is determined using the energy equivalent method, as shown in Figure 16. This method mainly uses the area enclosed by OC segment of the skeleton curve to be equal to the area of trapezoidal OACE segment to calculate the length of AC segment, thereby determining the yield displacement Δ*_y_*, and the vertical axis value corresponding to Δ*_y_* is the yield load *P_y_*. The maximum load that the specimen can withstand is *P_m_*, and the corresponding value when the maximum load drops to 85% is failure load *P_u_*. Δ*_m_* and Δ*_u_* are the maximum and failure displacement values corresponding to *P_m_* and *P_u_*, respectively. The ductility factor of the specimen is *u*, as shown in Equation (1).

(1) $$u=\Delta_{u}/\Delta_{y}$$

The calculation results of the characteristic load and ductility are given in Table 5. The ductility coefficient of RC0 was 1.50, while the ductility coefficients of TRC1–TRC3 were 6.00, 4.77, and 5.55, respectively. Compared with the new-type specimen, the ductility of RC0 was the worst, indicating that RC0 had the least ductility. This is due to the fact that RC0 consists of concrete and steel reinforcement, which has limited ability to resist deformation and is prone to undergo shear damage under external loading.

Compared with TRC1, the yield and maximum loads of TRC2 were higher, increasing by 21.2% and 18.4%, respectively, while the failure displacement and the ductility coefficient were smaller, which indicated that increasing the thickness of the steel plate wrapping the beam improved the initial stiffness of the specimen. However, due to the cracking of the weld seam, the mechanical properties of the specimen degraded significantly, and the deformation capacity rapidly decreased.

Compared with TRC2, the yield and maximum loads of TRC3 decreased by 4.6% and 6.4%, respectively, while the failure displacement and the ductility coefficient increased slightly, indicating that the thickness of connectors had limited effect on the load-bearing capacity, but could ensure the stability of the bearing capacity to a certain extent.

### 4.4. Rigidity Degeneration

Stiffness is an important index affecting the seismic performance of structures, and its stability is directly related to whether the structure can maintain a high bearing capacity and good deformation adaptability under seismic loads. The phenomenon of stiffness degradation reveals the gradually accumulated damage state of the structure during continuous stress. In this paper, the average stiffness Ki under the same loading cycle is used to represent the stiffness degradation of joints. The calculation formula is as follows:

(2) $$K_{i}=\frac{\left|+P_{i}\right|+\left|-P_{i}\right|}{\left|+\Delta_{i}\right|+\left|-\Delta_{i}\right|}$$

in which +*P_i_* and −*P_i_* are the positive and negative maximum loads under the *i*-th cycle load, while +Δ*_i_* and −Δ*_i_* are the displacements corresponding to +*P_i_* and −*P_i_*.

**Figure 17:**
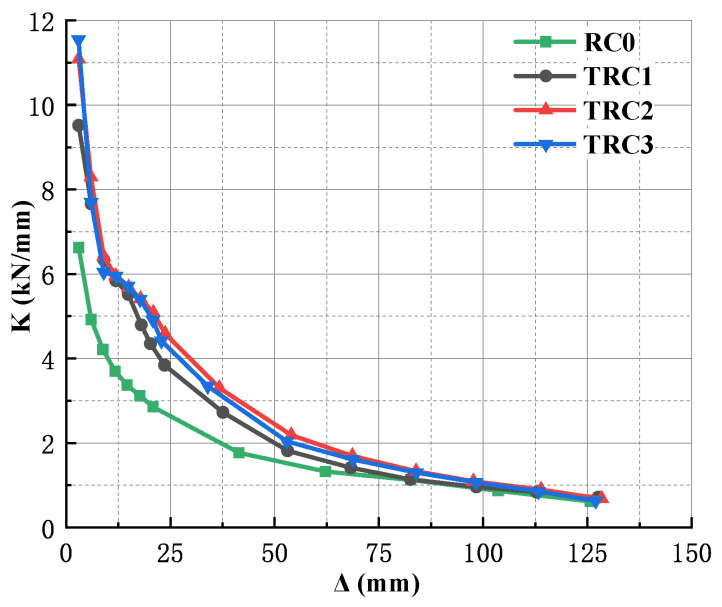
Rigidity degeneration of the specimens.

The results are shown in Figure 17, it can be concluded that:

(1) Compared with TRC1–TRC3, RC0 had smallest stiffness in the initial stage and fastest stiffness degradation rate. This is because that the elastic modulus of concrete is smaller than that of the steel. During the loading process, concrete is frequently affected by tensile and compressive forces, and the damage gradually accumulates, resulting in a smaller initial stiffness of the specimen and a faster decrease rate of stiffness. In contrast, before the cracking of the weld seam, the new-type specimens are mainly subjected to external loading by steel, so the rigidity can maintain a certain degree of stability before reaching the ultimate bearing capacity.

(2) For TRC1–TRC3, when the loading displacement was less than 15 mm, the initial stiffness of TRC1 was smaller, the initial stiffness of TRC2 and TRC3 were close to each other, and the stiffness degradation of each specimen was not significantly different. This is due to the fact that the thickness of the steel plate wrapping the beam of TRC1 is thinnest, which contributes relatively little to the overall stiffness of the specimen. At the same time, in the early stage of loading, the specimens are in the elastic stage, and the stress–strain relationship of materials is basically linear, so there is not much difference in the rigidity degradation of these three specimens.

When the loading displacement was between 15 and 84 mm, the rigidity degradation trend of the new-type specimen began to show differences with fastest rigidity degradation in TRC1 and slower stiffness degradation in TRC2 and TRC3. This is due to the fact that with the accumulation of damage in the specimen, TRC1 has a relatively weaker restraining effect on the concrete due to the thinner thickness of the steel plate, making it more prone to producing local damage and stress concentration during the stress process, thus accelerating the rigidity degradation.

When the loading displacement was greater than 84 mm, the rigidity degradation of TRC1 was slow, while that of TRC2 and TRC3 was slightly faster. When the ultimate displacement was reached, the curves of these three specimens were basically the same. This is because that the main damage to the specimen has already formed, and the part of the specimen bearing the external load is reinforcement concrete.

(3) Comparing the rigidity degradation curves of TRC2 and TRC3, it could be found that the rigidity degradation trends of these two specimens were almost coincident, which indicates that the thickness of the T-shaped steel plate had almost no effect on the rigidity degradation of the specimen.

### 4.5. Energy Dissipation Capacity

**Figure 18:**
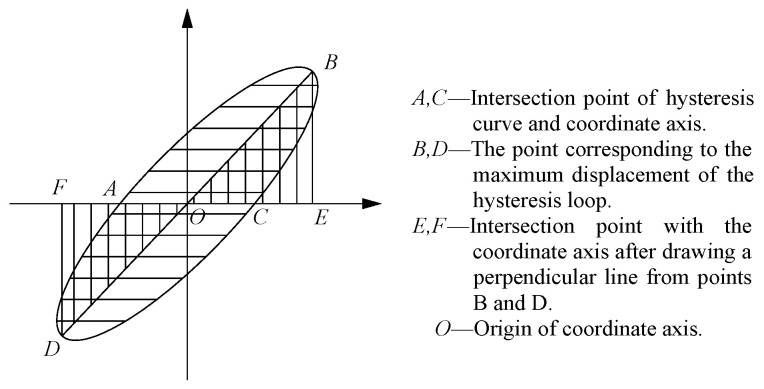
Calculation diagram of the equivalent viscous damping coefficient.

The area enclosed by the hysteresis curve of the specimen represents its energy dissipation capacity. The larger the area enclosed by the hysteresis curve at each level, the stronger the energy dissipation capacity of joint. This paper uses the equivalent viscous damping coefficient *h_e_* to represent the energy dissipation performance of the specimen. The larger *h_e_* is, the stronger its energy dissipation capacity. The calculation method of *h_e_* is given in Equation (1) and Figure 18.

(3) $$h_{e}=\frac{1}{2\pi }\cdot \frac{S_{ABCD}}{S_{OBE}+S_{ODF}}$$

where *S_ABCD_*, *S_OBE_*, and *S_ODF_* are the areas enclosed by the hysteresis curve, triangle *OBE*, and triangle *ODF*, respectively.

**Figure 19:**
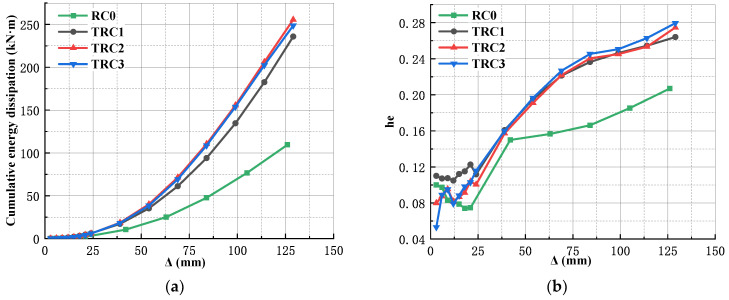
Energy dissipation of the specimens: (**a**) cumulative energy dissipation; (**b**) the equivalent viscous damping coefficient.

Figure 19a shows the cumulative energy dissipation curves of the specimen. From the figure, it could be seen that when the loading displacement was 25 mm, the cumulative energy dissipation of RC0 developed slower compared to that of TRC1–TRC3, indicating that the wrapped steel improved the rigidity and ductility of the specimen, which enhanced the energy dissipation capacity. Therefore, TRC1–TRC3 could absorb and dissipate more energy.

Compared with TRC1, the cumulative energy consumption of TRC2 increased by 8.37%, indicating that increasing the thickness of the steel plate wrapping the beam can improve the energy consumption capacity of the specimen. This is due to the fact that the wrapped steel, as a reinforcing layer, is able to carry more external loads and thus consume more energy. Compared with TRC3, the cumulative energy consumption of TRC2 increased by 2.75%, indicating that increasing the thickness of the T-shaped steel plate had little effect on improving the energy consumption capacity of the specimen. This is because that the damage to the specimen mainly appears in the weld seam of the wrapped steel on the column side, while the T-shaped steel plate does not undergo any damage.

Figure 19b gives the equivalent viscous damping coefficient curves of the specimen. From the figure, it could be observed that after the yielding of the specimen, the equivalent viscous damping coefficient of RC0 developed slowly compared to those of TRC1–TRC3. This is due to the reason that the RC0 mainly dissipates energy through the yielding of reinforcement bars and the cracking of concrete. When the specimen is in the elastic–plastic state, the energy dissipation ability gradually decreases with the cracking and spalling of concrete.

The development trend of the equivalent viscous damping coefficient of TRC2 and TRC3 was basically the same, which indicated that the thickness of the T-shaped steel plate had little effect on the equivalent viscous damping coefficient. Compared with TRC2, the initial equivalent viscous damping coefficient of TRC1 was larger, and the increase in this value was slightly lower than that of TRC2 after the specimen was loaded to the failure displacement, indicating that increasing the thickness of the wrapped steel was beneficial for improving the equivalent viscous damping coefficient.

## 5. Conclusions

In this paper, a new solution is proposed for the reinforcement and renovation of existing buildings. To ensure the integrity of the specimen, steel plates are wrapped around the column, the connectors are welded onto the steel plate, and the longitudinal bars of the beam are welded onto connectors. Then, low-cyclic loading tests are carried out on the specimens to analyze the seismic performance and comparison results with cast-in situ specimen with same size. The main conclusions are given as follows:

(1) Compared with cast-in situ specimen, the damage to the new-type specimen is mainly concentrated at the connection between the wrapped steel of the beam and column, as well as the weld seam of the wrapped steel of the column. The concrete, steel reinforcement, and T-shaped steel plate in the core area of the joint experience smaller forces and no damage phenomenon occurs. The load of the new-type specimen is mainly experienced by the steel plate and the weld seam before the failure of the specimen, and the mechanical properties of the specimen will decrease rapidly after the cracking of the weld seam.
(2) Compared with RC0, the bearing capacity of TRC1–TRC3 increased by −1.03%, 15.80%, and 3.26%, respectively. At the same time, it shows significant advantages in ductility, rigidity degradation, and energy-consuming capacity, which indicates that the new-type specimen can consume more energy during the earthquake process and has better deformation capacity and stability in stiffness throughout the entire failure process. This is due to the fact that the main load-bearing part of new-type specimen is the wrapped steel before reaching the maximum bearing capacity, while the damage to concrete in RC0 under repeated loading will gradually accumulate. The mechanical performance of the steel is more stable, so the overall seismic performance of the new-type specimen is better.
(3) Compared with TRC1, the load-carrying capacity and cumulative energy consumption of TRC2 increase by 17.01% and 8.37%, respectively, and the ductility coefficient decreases by 20%. This phenomenon indicates that increasing the thickness of the wrapped steel of the beam can effectively improve the load-bearing capacity and energy consumption capacity of the specimen. However, due to the increase in the thickness of the steel plate, the stiffness of the specimen increases, and the load-bearing capacity and energy consumption capacity can be maintained at a higher level before reaching the maximum load, but the ductility becomes weaker after the cracking of the weld seam.
(4) Compared to TRC1, the load-bearing capacity decreases by 4.7%, the ductility coefficient increases by 16.4%, and cumulative energy dissipation decreases by 2.7, indicating that the thickness of the T-shaped steel plate has little effect on the seismic performance of the specimen. This is due to the fact that before the failure of the specimen, the main load-bearing part is the wrapped steel, and the load borne by the reinforced concrete part is very small, and the T-shaped steel plate is also subjected to less stress.
(5) Taking into account the seismic performance of various specimens analyzed in this paper, it can be concluded that the design parameters of TCR2 are optimal. In engineering applications, the influence of the thickness of the steel plate wrapped around the beam should be considered first, and then the amount of the steel used should be increased as much as possible.

The research results of this paper are helpful in promoting the development of reinforcement and renovation of existing buildings. However, due to the limitations of research time, only the influence of the thickness of the T-shaped steel plate and the wrapped steel of the beam was analyzed. Further in-depth research is needed on the factors such as specimen size, steel plate strength, and concrete strength to comprehensively understand the mechanical characteristics of the new-type specimen. At the same time, considering that the failure of specimens is mainly concentrated at the weld seam of the wrapped steel plate, further research is needed on the influence of weld grades and other structural measures, so as to improve the stress performance of joints and enhance the safety of the structure.

## Figures and Tables

**Table 1:** The parameter and arrangement of materials.

Component	Size of Cross-Section (mm)	The Parameter and Strength Grade of Longitudinal Reinforcement	The Parameter and Arrangement of Lateral Reinforcement	Strength Grade of Concrete (MPa)
Beam	200 × 400	C16	A6@50/100	40
Column	300 × 300	C20	A6@50/75	50

**Table 2:** Design parameters of the specimen.

Number	Length/mm		Length of Encased Steel Plate (mm)		Dimensions of T-Shaped Steel Plate (mm)		Thickness of Encased Steel Plate (mm)	
	Beam	Column	Beam	Column	Length	Thickness	Beam	Column
RC0	1400	2000	-	-	-	-	-	-
TRC1	1400	2000	750	1500	200	5	2	10
TRC2	1400	2000	750	1500	200	5	3	10
TRC3	1400	2000	750	1500	200	3	3	10

**Table 3:** Compressive strength of concrete.

Concrete Type	C40	C50	C40-1
Compressive strength (MPa)	48.3	56.1	46.6

**Table 4:** Strength of the steel.

Steel Types	Strength Grade	Thickness (Diameter) (mm)	Yield Strength (MPa)	Ultimate Strength (MPa)	Elastic Modulus (10^5^ MPa)
Steel bars	HPB300 (A6)	6	382	536	2.10
	HRB400 (C16)	16	464	634	2.06
	HRB500 (C20)	20	572	736	2.06
Steel plates	Q345	2	346	459	2.06
	Q345	3	420	503	2.06
	Q345	5	333	444	2.06
	Q345	10	359	425	2.06

**Table 5:** Characteristic load and ductility factor.

Number	Loading Direction	Point Of Yield Load		Point of Maximum Load		Point of Failure Load		Ductility Factor
		$P_{y}$/kN	$\Delta_{y}$/mm	$P_{m}$/kN	$\Delta_{m}$/mm	$P_{u}$/kN	$\Delta_{u}$/mm	$u=\Delta_{u}/\Delta_{y}$
RC0	Push	106.87	74.40	111.94	82.64	95.15	120.67	1.62
	Pull	74.92	89.71	75.29	103.96	64.00	124.78	1.39
	Average	90.90	82.06	93.62	93.30	79.58	122.73	1.50
TRC1	Push	97.85	20.14	110.79	37.06	94.17	112.46	5.58
	Pull	76.05	19.76	94.15	38.15	80.03	126.98	6.43
	Average	86.95	19.95	102.47	37.61	87.10	119.72	6.00
TRC2	Push	114.71	23.44	129.63	38.15	110.19	92.04	3.93
	Pull	96.02	20.15	113.02	35.39	96.07	116.03	5.76
	Average	105.37	21.80	121.33	36.77	103.13	104.04	4.77
TRC3	Push	105.06	19.23	115.59	34.24	98.25	113.53	5.90
	Pull	96.07	21.49	111.44	33.65	94.72	112.36	5.23
	Average	100.57	20.36	113.52	33.95	96.49	112.95	5.55

## Data Availability

The original contributions presented in this study are included in this article, and further inquiries can be directed to the corresponding author.

## References

[1] Hui W.F. (2021). Study on Adding Floors and Strengthening of a Certain Multi-Story Reinforced Concrete Frame Structure. Master’s Thesis.

[2] Zhang X., Li A.Q., Zhao K.Z. (2011). Advances in assessment and retrofitting of building structures. Eng. Mech..

[3] Zhang X., Yue Q.X. (2021). Development on theory and technology on the evaluation, strengthening and retrofitting of existing structures. J. Shandong Jianzhu Univ..

[4] Nouri F., Valipour H.R. (2019). Semi-rigid partial-strength steel-timber composite (STC) connections with mechanically anchored steel rods. J. Constr. Steel Res..

[5] Nouri F., Valipour H.R. (2020). Moment-rotation model for steel-timber composite connections with slab continuity steel rods. J. Constr. Steel Res..

[6] Wiktor R. (1994). Glulam Connections Using Epoxy Glued-in Rebars. Master’s Thesis.

[7] Ozturan T., Ozden S., Ertas O. (2006). Ductile connections in precast concrete moment resisting frames. PCI J..

[8] Lee D.J., Lee J.D., Oh T., Kang T. (2014). Seismic experiment of precast concrete exterior beam-column joint using bolt type connection and prestressing method. J. Korea Concr. Inst..

[9] Baran E., Mahamid M., Baran M., Kurtoglu M., Torra-Bilal I. (2021). Performance of a moment resisting beam-column connection for precast concrete construction. Eng. Struct..

[10] Ding K., Ye Y., Ma W. (2021). Seismic performance of precast concrete beam-column joint based on the bolt connection. Eng. Struct..

[11] Tankut T., Korkmaz H.H. (2005). Performance of a precast concrete beam-to-beam connection subject to reversed cyclic loading. Eng. Struct..

[12] Rodríguez M.E., Torres M.M. (2014). Seismic behavior of a type of welded precast concrete beam-column connection. PCI J..

[13] Magliulo G. (2017). Cyclic shear tests on RC precast beam-to-column connections retrofitted with a three-hinged steel device. Bull. Earthq. Eng..

[14] Esmaeili J., Ahooghalandary N. (2020). Introducing an easy-install precast concrete beam-to-column connection strengthened by steel box and peripheral plates. Eng. Struct..

[15] Huang W., Hu G.X., Zhang J.R. (2022). Experimental study on the seismic performance of new precast concrete beam-column joints with replaceable connection. Structures.

[16] Li Z.Y., Kang S.B., He H., Lu W.Q., Liu H.J., Lu C.J. (2023). Seismic behaviour of precast concrete beam-column connections with bolted end plates. Structures.

[17] Zhang Y., Ma W., Dai Y.T., Li K. (2024). Seismic performance verification and theoretical stiffness calculation for a novel assembled concrete dry-connected beam-column node. Structures.

[18] Vandoros K.G., Dritsos S.E. (2008). Concrete jacket construction detail effectiveness when strengthening RC columns. Constr. Build. Mater..

[19] Guo R.H. The reliability of enlarging the cross-section in reinforcing the frame construction buildings. Proceedings of the ASME 2011 International Manufacturing Science and Engineering Conference.

[20] Martin V., Peter K., Ashot T. (2018). Numerical analysis of strengthening concrete columns by high performance fiber concrete. MATEC Web Conf..

[21] Kim C.S., Park H.G., Chung K.S., Choi I. (2014). Eccentric axial load capacity of high-strength steel-concrete composite columns of various sectional shapes. J. Struct. Eng..

[22] Kim S.B., Lee E.T., Kim J.R., Kim S.S. (2016). Experimental study on bending behavior and seismic performance of hybrid composite beam with new shape. Int. J. Steel Struct..

[23] Xu C.X., Peng P., Deng J., Wan C.C. (2018). Study on seismic behavior of encased steel jacket-strengthened earthquake-damaged composite steel-concrete columns. Int. J. Build. Eng..

[24] Chao C.G., Ha G.J., Kang H.W., Feo L. (2013). Seismic improvement of RC beam-column joints using hexagonal CFRP bars; combined with CFRP sheets. Compos. Struct..

[25] Beydokhti E.Z., Shariatmadar H. (2016). Strengthening and rehabilitation of exterior RC beam–column joints using carbon-FRP jacketing. Mater. Struct..

[26] Truong G.T., Dinh N.H., Kim J.C., Choi K.K. (2017). Seismic Performance of Exterior RC Beam–Column Joints Retrofitted using Various Retrofit Solutions. Int. J. Concr. Struct. Mater..

[27] Azarm R., Maheri M.R., Torabi A. (2017). Retrofitting RC Joints Using Flange-Bonded FRP Sheets. Iran. J. Sci. Technol. Trans. Civ. Eng..

[28] Campione G. (2012). Strength and ductility of RC columns strengthened with steel angles and battens. Constr. Build. Mater..

[29] Tarabia A.M., Albakry H.F. (2014). Strengthening of RC columns by steel angles and strips. Alex. Eng. J..

[30] Pudjisuryadi P., Tavio, Suprobo P. (2015). Performance of square reinforced concrete columns externally confined by steel angle collars under combined axial and lateral load. Procedia Eng..

[31] Adibi M., Marefat S.M., Esmaeily A., Arani K.K., Esmaeily A. (2017). Seismic retrofit of external concrete beam-column joints reinforced by plain bars using steel angles prestressed by cross ties. Eng. Struct..

[32] (2019). Standard for Test Methods of Concrete Physical and Mechanical Properties.

[33] (2010). Metallic Materials-Tensile Testing-Part 1: Method of Test at Room Temperature.

[34] (2015). Specification for Seismic Test of Buildings.

